# Use of an Enactive Insole for Reducing the Risk of Falling on Different Types of Soil Using Vibrotactile Cueing for the Elderly

**DOI:** 10.1371/journal.pone.0162107

**Published:** 2016-09-07

**Authors:** Martin J. -D. Otis, Johannes C. Ayena, Louis E. Tremblay, Pascal E. Fortin, Bob-Antoine J. Ménélas

**Affiliations:** 1 Department of Applied Sciences, University of Quebec at Chicoutimi (UQAC), REPARTI centre, Chicoutimi, Quebec, Canada; 2 Department of Health Sciences, University of Quebec at Chicoutimi, Chicoutimi, Quebec, Canada; 3 Department of Electrical Engineering and Computer at the Center for intelligent machine, McGill University, Montreal, Quebec, Canada; 4 Department of Mathematics and Computer Sciences, University of Quebec at Chicoutimi, Chicoutimi, Quebec, Canada; Shanghai Jiao Tong University, CHINA

## Abstract

**Background:**

Our daily activities imply displacements on various types of soil. For persons with gait disorder or losing functional autonomy, walking on some types of soil could be challenging because of the risk of falling it represents.

**Methods:**

In this paper, we present, in a first part, the use of an enactive shoe for an automatic differentiation of several types of soil. In a second part, using a second improved prototype (an enactive insole), twelve participants with Parkinson’s disease (PD) and nine age-matched controls have performed the Timed Up and Go (TUG) test on six types of soil with and without cueing. The frequency of the cueing was set at 10% above the cadence computed at the lower risk of falling (walking over the concrete). Depending on the cadence computed at the lower risk, the enactive insole activates a vibrotactile cueing aiming to improve gait and balance control. Finally, a risk index is computed using gait parameters in relation to given type of soil.

**Results:**

The frequency analysis of the heel strike vibration allows the differentiation of various types of soil. The risk computed is associated to an appropriate rhythmic cueing in order to improve balance and gait impairment. The results show that a vibrotactile cueing could help to reduce the risk of falling.

**Conclusions:**

Firstly, this paper demonstrates the feasibility of reducing the risk of falling while walking on different types of soil using vibrotactile cueing. We found a significant difference and a significant decrease in the computed risks of falling for most of types of soil especially for deformable soils which can lead to fall. Secondly, heel strike provides an approximation of the impulse response of the soil that can be analyzed with time and frequency-domain modeling. From these analyses, an index is computed enabling differentiation the types of soil.

## Introduction

Falls are an important cause of morbidity and mortality in elder people. Approximately one-third of community-dwelling adults aged over 65 years experience at least one fall every year and 75% of people with Parkinson’s disease (PD) are subject to an increased risk of falling [1]. Because of the problems that they lead to, many programs have been created in order to prevent accidental falls. As pointed out by Filiatrault et al. [2], to be effective, these programs have to target multiple factors that contribute to the risk of falling. In this state of mind, several programs have coupled the practice of physical exercises to the analysis of balance, postural instability and gait. Previous research efforts have focused on vision control, hearing and blood pressure while others have taken all these factors and several others into account [3]. Even though noticeable advancements in this domain, it seems that no program has yet offered an on-site assistance to the user while considering his environment. We think that recent technological achievements can be exploited in order to assist a frail user such as a person with PD in situations that can represent a certain risk of falling. To achieve this goal, one of the first requirements is to perform the automatic differentiation of several types of soil which are part of the user’s environment.

This idea comes from the domain in mobile robotic where analysis of terrains is crucial for autonomous control and decision making. Weiss et al. [4] concluded that numeric vision and vibration of the robot’s chassis are combined to decide whether the land can be safely crossed or not. In the same way [5], vibration analysis is coupled with a method labeled Terrain Input Classification on a powered electric wheelchair, to improve the control of the wheel. In locomotion, legged robots also use classification of terrain for control gait adaptation [6]. In the same order of ideas, loping body motion (i.e. gait bounce) is used with a limb/terrain interaction model for terrain discrimination [7]. Hoepflinger et al. [8] used multiclass AdaBoost classifier for terrain shape (concave and convex) and terrain surface (abrasive paper) classification implemented in a quadruped robot. However, the above mentioned methods are not adapted for terrain discrimination in the case of a human walker. Then, our research work proposes an enactive shoe that will help at discrimination physical properties of the soil. As mentioned previously, this effort is one the first requirements for the realization of on-site assistance aimed at preventing accidental falls.

The prevention of falls represents a major challenge which has been widely studied on frail elderly and pathological population in the past years [9]. The falls induced a kinesiophobia (fear of falling) by reducing the functional autonomy. Since the research works in this study focuses on a method to avoid falls, it is therefore necessary to compute a risk of falling level in healthy elderly and PD subjects which represents a potential threat to a fall. Here, we analysis in the literature: 1) the evaluation regarding a risk of falling while covering a clinical test such as the Timed Up and Go (TUG) and 2) the use of enactive shoe or insole in the prevention of accidental falls.

### Risk of falling evaluation

Previous research efforts showed that the risks of falling depend on several factors such as: (1) variations in measured parameters of gait, (2) the environment mainly characterized by the type of soil on which the user is walking, (3) human factor (awareness of environment), and (4) neurological deficits. The next sections briefly describe two of these factors to select an appropriate method of computing the risk of falling embedded in a microcontroller.

The first factor related to our work regarding the evaluation of a risk of falling concerns the gait parameters: Hamacher et al. [10] concluded that linear variability of temporal measures of stride, swing and stance time are the most significant parameters in distinguishing between fallers and non-fallers. An implementation model of this risk computation was successfully demonstrated by Noshadi et al. [11]. However, these studies do not consider the environment of the walker. Indeed, the variability of the gait parameters was previously demonstrated as a function of the soil properties and footwear [12] and also for low friction walkway [13] and movable platform [14]. It is also known that the type of soil can affect the gait [15]. Moreover, some studies relate the effects of unstable surfaces such as rocks [16] and hill transitions [17] on the gait parameters. The walker’s environment has a significant impact on the risk of falling. The first risk to assess in the user’s environment is the type of soil. It appears that a soil differentiation algorithm will allow us to evaluate potentially dangerous situations inside the walker’s environment. The second factor concerns the human factors: When an analysis is only conducted on the gait regardless of the surrounding environment, it is necessary to determine the current activity of the walker and its associated normal parameters fluctuations. In this particular situation, it should be possible to calculate a risk of falling level when parameters deviate from a precomputed normal trend. The design of such an algorithm for all daily activities is still an undergoing issue. However, there is a more straightforward way to analyze the risk of falling level: human factors. These factors include, among others, visual issues for perception of falling hazards [18]. This issue comes from the difference between the current measurements of friction and the psychophysical perception of friction [19]. In fact, it is known that a walker can perceive some material properties under the foot while walking [20]. Tactile information like vibrations may be used to perceive material properties such as texture [21], roughness, compliance and friction [22]. However, vision and audition could influence on the tactile perception and any coarse evaluation of a slippery surface could increase the risk of falling [23, 24]. Others factors such as cognitive or attentional process have not been considered in this paper.

The ability of the user to maintain balance in ordinary daily living activities is often evaluated by the Berg Balance Scale [25], Tinetti Balance Assessment Tool [26] or Timed Up and Go test [27]. In the next subsection, we covered the clinical test used in this study: the Timed Up and Go test.

### Timed up and go test (TUG)

Previously named the “Get-up and Go test”, the Timed Up and Go (TUG) test modifies the original test by adding a timing component to performance. The TUG test measures mobility in elderly people [27, 28] and is considered as a reliable tool for quantifying not only the locomotor performance but also the mobility among persons with PD [29–31]. This test requires an individual to stand up from a chair, walk three meters, turn around (180 degrees), walk back to the chair, and sit down again. In normal conditions, the researchers hypothesize that neurologically sound adults who are independent in balance and mobility skills are able to perform the test in less than 10 seconds. Participants who take more than 16 seconds to complete the test are associated with increased risk of falling in the activities of daily living [32].

Since several factors are related to balance issues and can lead to fall, this paper suggests using the physical properties of the soil and an individual aid implemented in the insole of a shoe to enhance perception of falling hazards and maintain balance. In past works, our laboratory designed a serious game which used an enactive insole in order to train balance of walker [33]. What follows reviews the enactive insoles/shoes that have been proposed in the past and also the use of cueing.

### Enactive insole or shoe for gait improvement

Typical enactive shoes use the sensors inside the sole to analyze the user’s gait. As shown by Magaa et al. [34], over the past decade, several types of shoes or insole featuring data acquisition and/or vibration transmission capabilities have been developed. Instrumented shoes with wireless capabilities demonstrated the feasibility of walking parameters computation such as heel-strike, toe-off, foot orientation and position [35]. Some factors associated to a risk of falling were analyzed and a risk factor index was computed with eight walking parameters such as pressure correlation, step time, cadence and stance-to-swing ratio [11]. Recently, a smartphone-based system has proved to be an effective tool for showing clinical tests parameters at home [36]. Indeed, a smartphone software with instrumented shoe have been used to provide on-site assistance to a user using a serious gaming [37] and to train balance over different types of soil [33]. However, much of these works have been aimed at rendering symbolic information (i.e. directional indicator, encoded message) rather than conveying ecological stimulus [38]. In the field of interactive shoes, an instrumented shoe was proposed by Paradiso et al. [39] for gait characteristics acquisition while being enabled with a sound feedback for dance performances. Samsung Electronics was also interested in dance training with a patent presented by Kim et al. [40]. Recently, an instrumented shoe dedicated to analyzing and maintaining the balance via a single-frequency vibrotactile feedback was presented by Shieh et al. [41].

In this line of thoughts, other studies demonstrated that improvements of spatiotemporal gait pattern can be obtained with the use of an appropriate stimulation such as auditory, visual or vibrotactile cueing [42]. In this paper, we consider the case where only a rhythmic vibrotactile is sent back to the user. Previous studies proposed the use of a vibrating neurofeedback system [43, 44] or a miniature vibrating apparatus [45], that were composed of sensor and stimulation components. The sensor elements consisted in an inertial measurement unit placed at shank to detect freezing of gait (FOG) episodes with the embedded time-frequency analysis algorithm. The stimulation part was a vibrator pad placed below the lateral malleolus, to facilitate lateral weight shift during FOG. Their results showed that the real-time somatosensory cue could help gait re-initiation by facilitating lateral weight shift during FOG. The shortened FOG duration decreased the turning time in people with PD. However, the effects of somatosensory cueing in their work have been reported only in a gait initiation task and were only reported to improve the timing and movement outcome of gait initiation. Their evaluation did not take into account everyday life activities, medication or environmental perturbations of the user. These parameters are well known in the study of walking balance and are discussed by Ganz et al. [1].

In the light of all this, to the best of our knowledge, no study has investigated the impact of types of soil and an appropriate cueing in the computation of risk of falling. Our research addresses this throughout an enactive shoe that let to determine the type of soil that a person is walking on. For this, we analyze vibrations and forces under the feet and use these data to discriminate soil physical properties. The vibrations of the soil are acquired from each heel strike performed in a first experiment. After the differentiation of the terrain, if a certain level of risk is detected (such as presence of a top-layer of water on ice), an appropriated stimulus could be conveyed fastly to the user. The stimulus may be vibrotactile [46], mechanical (adaptation of the sole stiffness in function of the activity [47]) or variation of the adherence or friction under the sole [48]. In the same way, in the case of cadence decrease, a rhythmic vibrotactile feedback can be rendered. Here, in a second experiment, we investigate the use of a rhythmic vibrotactile feedback to help person with PD at maintaining their cadence. The rhythmic vibrotactile is sent at a frequency of 10% above the cadence computed over the lowest risk soil (the concrete). The choice of haptic (vibrotactile) as the mean of communication is based on the fact that elderly often can have hearing and/or visual problems and probably peripheral sensory impairment like proprioception [49]. Moreover, the haptic channel seems to be quite appropriate for communicating information since it offers a suitable medium and more safety when recipients are engrossed in a primary visual and/or auditory task [50]. In addition, the vibrotactile feedbacks have repeatedly demonstrated their effectiveness in correcting sway and balance of walkers [46, 51–56].

## Materials and Methods

In spite of many remarkable achievements, fall prevention programs currently fail to provide on-site assistance to users. Smartphones have received a lot of attention in the domain of home physiognomic monitoring. Among other things, they are now employed in public health surveillance through wireless health sensors [57] and can be used in telemedicine (emergency health care, intensive care patients monitoring and home telecare) [58]. This trend promotes the presence of a mobile interface combined to the smartphone in order to provide personalized assistance in real-time. Our proposed system is centered around an enactive device driven by a real-time system running on a smartphone. This device will serve for gait disorder analysis and for long term monitoring of persons in loss of mobility.

### Electronic hardware of the enactive shoe

**Fig 1:**
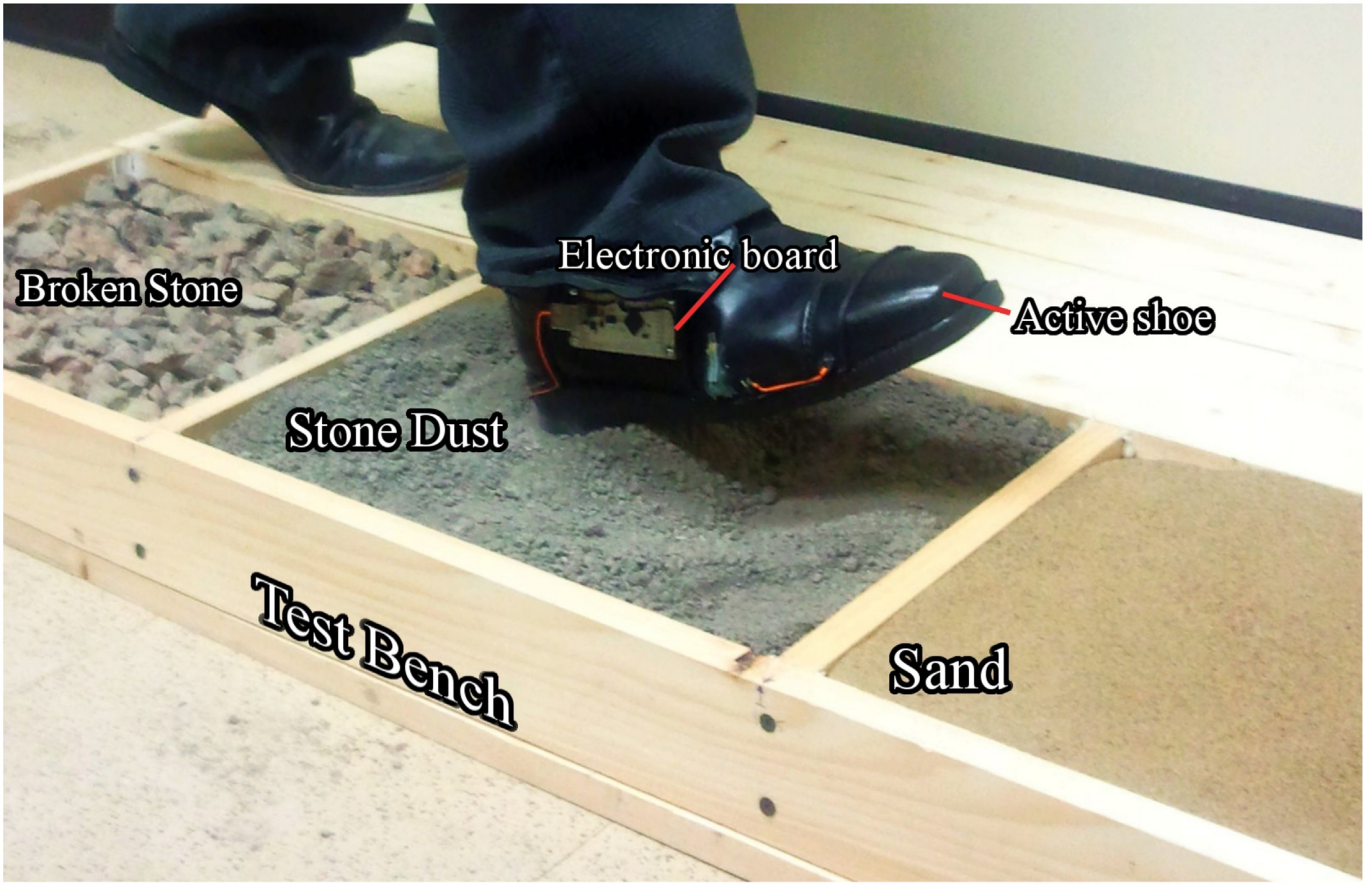
Enactive shoe prototype.

**Fig 2:**
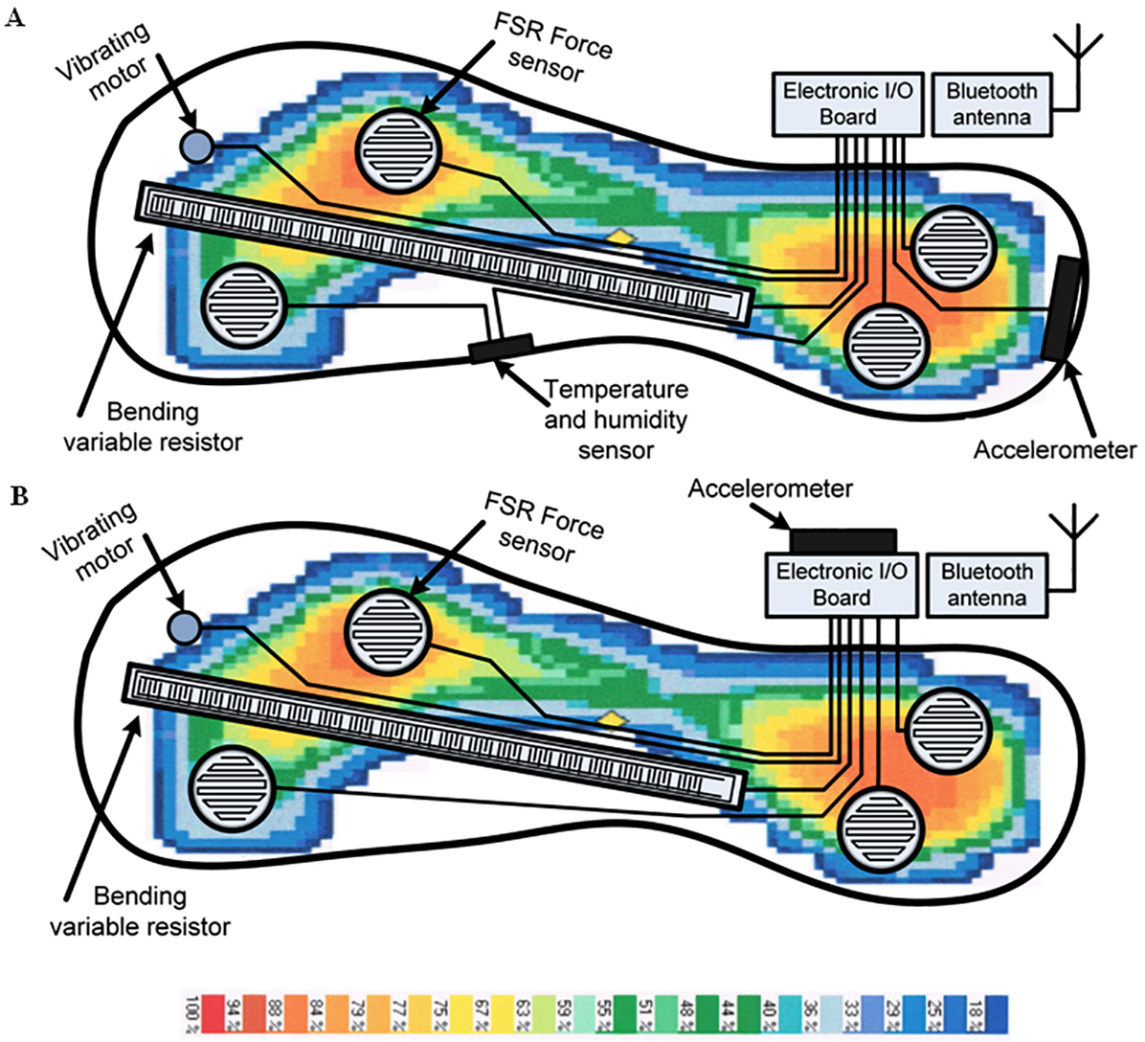
Location of the sensors and the actuator. (A) the sole of the enactive shoe. (B) the enactive insole.

The enactive shoe (Fig 1) employed in this project contains an actuator (vibrating motor) and several sensors such as a three-axis accelerometer, FSR force sensors and a bending variable resistor. All these components are located in the sole of the enactive shoe. A schematic of this sole is presented in Fig 2A. The Fig 2B also presents a schematic of the enactive insole used. In order to characterize physical properties of the environment like vibrations of the soil at heel strike, the accelerometer and the force sensors help measuring vibrations at heel strike. In contrast with terrain classification achieved by a rover (in mobile robotic application) where the accelerometer is located in the frame of the robot such as presented in [59], the accelerometer in this enactive shoe is located directly between the force applied by the whole human body and the soil inside the rigid part of the heel (Fig 2A). In consequence, the scope of this study is limited to the analysis of heel strikes. Any other interactions (such as movement at the surface of the soil) will not be considered for this research work. The bending variable resistor measures the deformation of the sole in order to acquire more insights during the propulsion phase of the gait. These sensors are mainly used to evaluate a risk of falling leading to an activation of the vibrotactile actuators as a biofeedback cueing. Indeed, the vibrotactile actuator is activated to introduce a rhythmic walking or when the risk increases over a reference such as a normal walking on the concrete (baseline) or any other similar soil.

**Fig 3:**
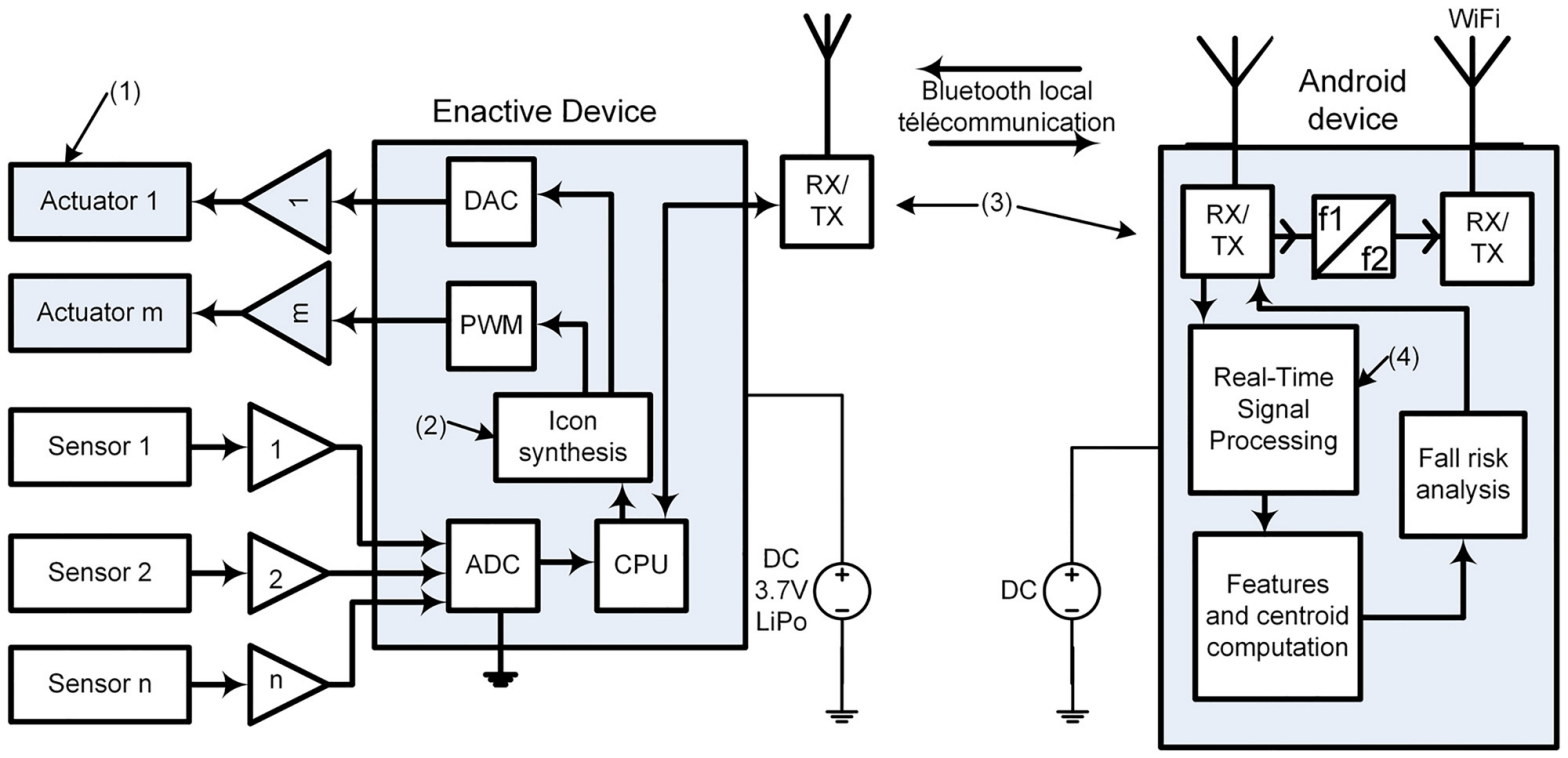
Electronic hardware of the two devices used in this study.

The sensors of the enactive shoe or the enactive insole are acquired using the electronic board shown in Fig 3. It contains an analogic to digital converter (ADC) and has Bluetooth capabilities. The microcontroller embedded in the device is a PIC 24 (16 bits architecture) from Microchip. The risk level should be computed by the PIC microcontroller and transmitted via Bluetooth (local telecommunication) to an Android Smartphone where the data is logged and analyzed in real-time. More details about the electronic hardware and operations are provided in the paper [60].

A preprocessing algorithm is applied on the acceleration waveforms acquired at each heel strike before the differentiation of soil physical properties and the computation of a risk of falling. If both the soil properties and the gait are found to be potentially dangerous and could be lead to a risk of falling, a signal is sent back to the walker which uses an icon synthesis algorithm to transmit adequate rhythmic vibrotactile cues to the walker. Each vibrating actuator is activated by a Pulse Width Modulation (PWM) signal using a rhythmic pattern. The rhythmic pattern is computed using 10% above the cadence of the walker at the instant before the risky situation. The 10% above the cadence is used to increase the difficulty of walking, the capacity of decoding sensory messages for central nervous system and probably to pay more attention (cognitive) at the stimulation. The ideal frequency of stimulation has yet to be fully defined, but it is known that auditory cues ranging from 90% to 125% of preferred cadence have shown benefit in terms of gait velocity [61–64], stride length [61, 64–66] and cadence [61–64, 66]. In addition, Moreau et al. [67] who used higher auditory frequencies (20% and 40% above the preferred walking cadence) have found an increased of freezing of gait in PD subjects. Then, the use of 10% in this study is based on frequency suggested by the literature.

### Automatic differentiation of types of soil using the enactive shoe

Based on works regarding terrain analysis in mobile robotic, in this section we use the accelerometer inserted in the heel of the enactive shoe in order to differentiate several types of soil. The acceleration of the heel is recorder at a sampling frequency around 1KHz. After that, the type of soil can be associated to an index enabling its differentiation. In the second experiment, using the enactive insole, it will be possible to associate a risk of falling for each type of soil and then try to reduce this risk using a vibrotactile cueing.

#### Experimental conditions

Hypothesis: We hypothesize that the accelerometer inserted in the heel of the shoe can serve for the differentiation of several types of soil. The physical properties of the soil should be reflected in terms of vibrations that occur between the sole of the shoe and the soil. Therefore, using the accelerometer, we should be able to measure the difference of the physical properties. In other words, using our enactive shoe, we will be able to differentiate from soils having different physical properties. Six types of soil were used for this first part of the experiment.

Types of soil: For an efficient measurement of the soil’s reaction, the heel should have nearly the same stiffness as the harder material to identify. In other case, the measurement will be coupled with the elastic deformation of the heel (equivalent stiffness and damping of the heel). We choose to compare five granular soil types: broken stone, stone dust, sand, ice and snow; which can be considered as the same soil class. To analyze the effect of the heel, we compare these granular soils with concrete. Indeed, concrete may be seen as having infinite inertia with a non-dominant time-response compare to the heel’s time-response.

Experimental procedure: The man for who the shoe had been designed is aged of 36 years old, 72 Kg and has no gait disorder. He wore the shoe for the experiment. On each of the six types of soil, he achieved thirteen steps one after the other (see Fig 1). During this experiment, for each step, data coming from the accelerometer and the force sensors were recorded and transmitted using the Bluetooth communication link.

#### Data measured from the enactive shoe

**Fig 4:**
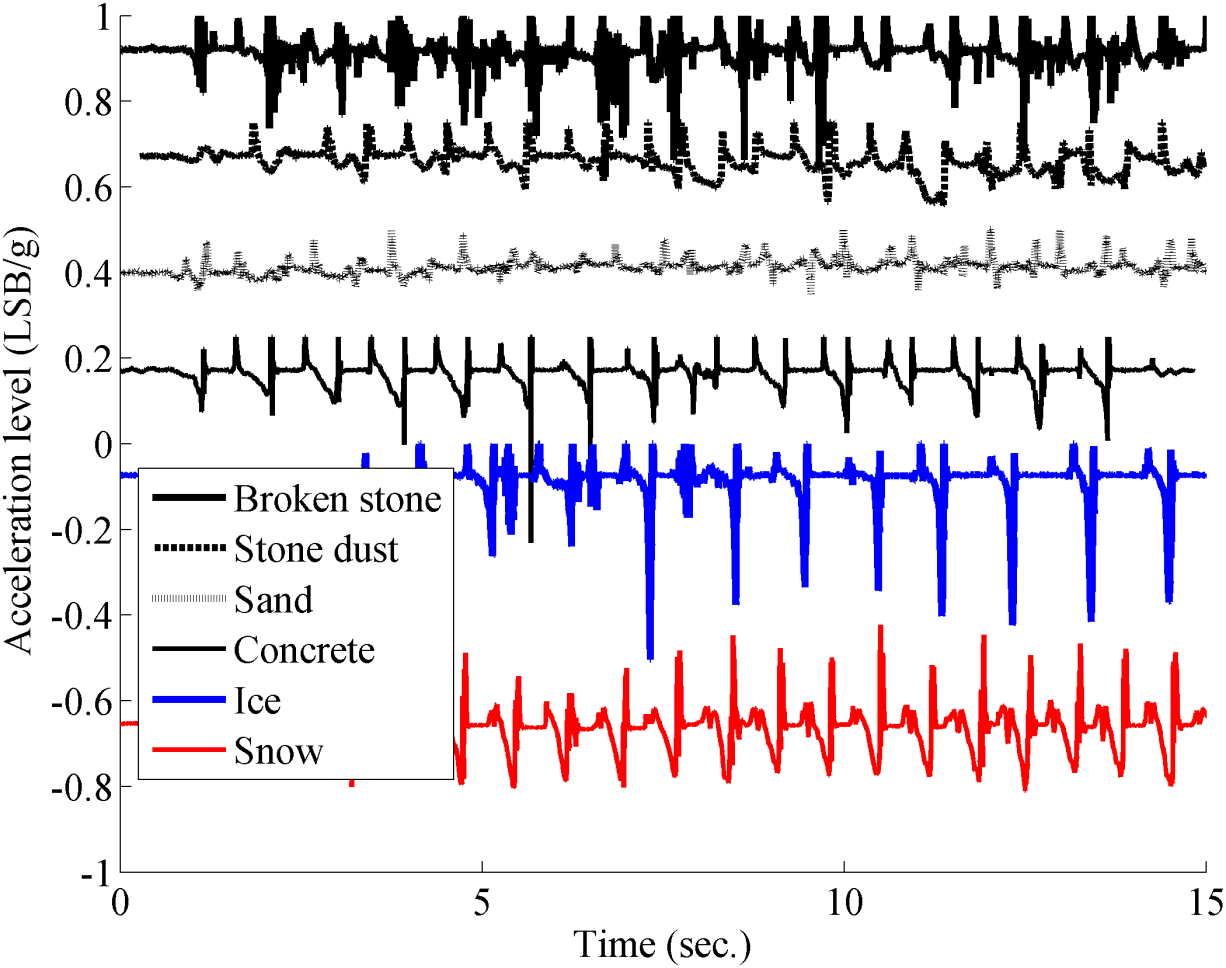
Thirteen heel strikes and acceleration measurements of the soil vibration.

Fig 4 shows the acceleration logged for the six types of soil. Visual inspection indicates a noticeable difference between the graphs. This difference is explained by the fact that data (the vibrations) measured by the accelerometer represent the variation of the impact force between the shoe and the soil. These variations are related to the physical properties of the soil. For the deformable soils (composed of multiple grains) these properties are characterized by different parameters. For example one can quote: the size of a grain and its geometry, the grain density (space available between the grains) and the corresponding rheological model of the soil. These physical properties allow the grains to move when the foot applies a force. During movement of the soil, the friction between these grains generates vibrations. Therefore, these vibrations are a time-response of the physical properties of the granular soil excited by the applied force. These vibrations during the heel contact, which is similar to an impact response, are measured by the accelerometer and are shown in Fig 4. All this explains the differences observed between the five other graphs. As opposed to the others, the concrete is a non-deformable soil. We thus understand that the vibrations corresponding to the impact with this model are different from the previous ones. These measurements come from the same accelerometer in the Z-axis located in the sole. The offset on each measurement for Fig 4 is only to facilitate the reading of each separate signal. From this database (data collected during the test), we suggest an automatic process embedded in the microcontroller that will help us at automatically differentiating these types of soil. This treatment counts two main steps. After the differentiation, a corresponding risk is computed and then a rhythmic vibrotactile cueing is sent to the walker with the help of the actuator located in the insole.

**Fig 5:**
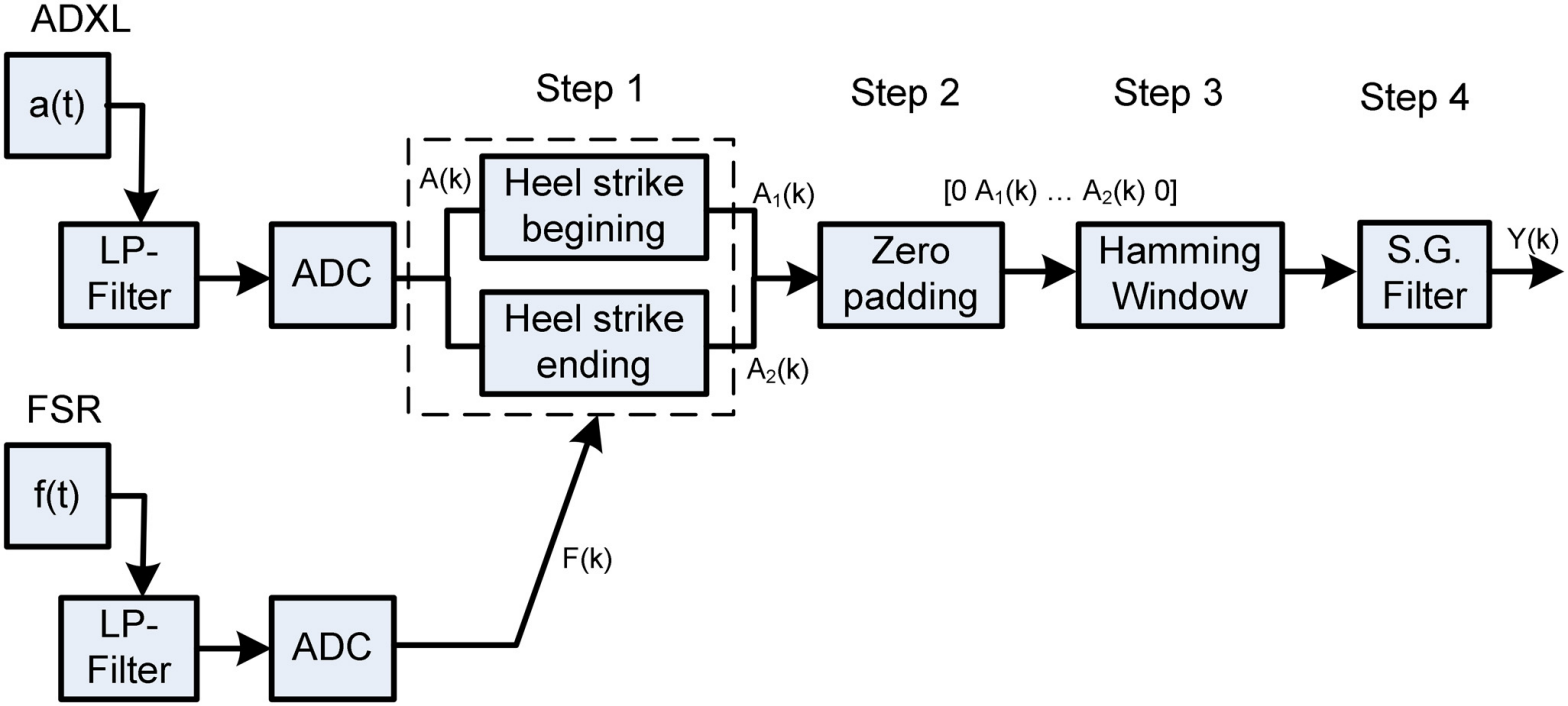
Segmentation of the acceleration signal with signal filtering.

**Fig 6:**
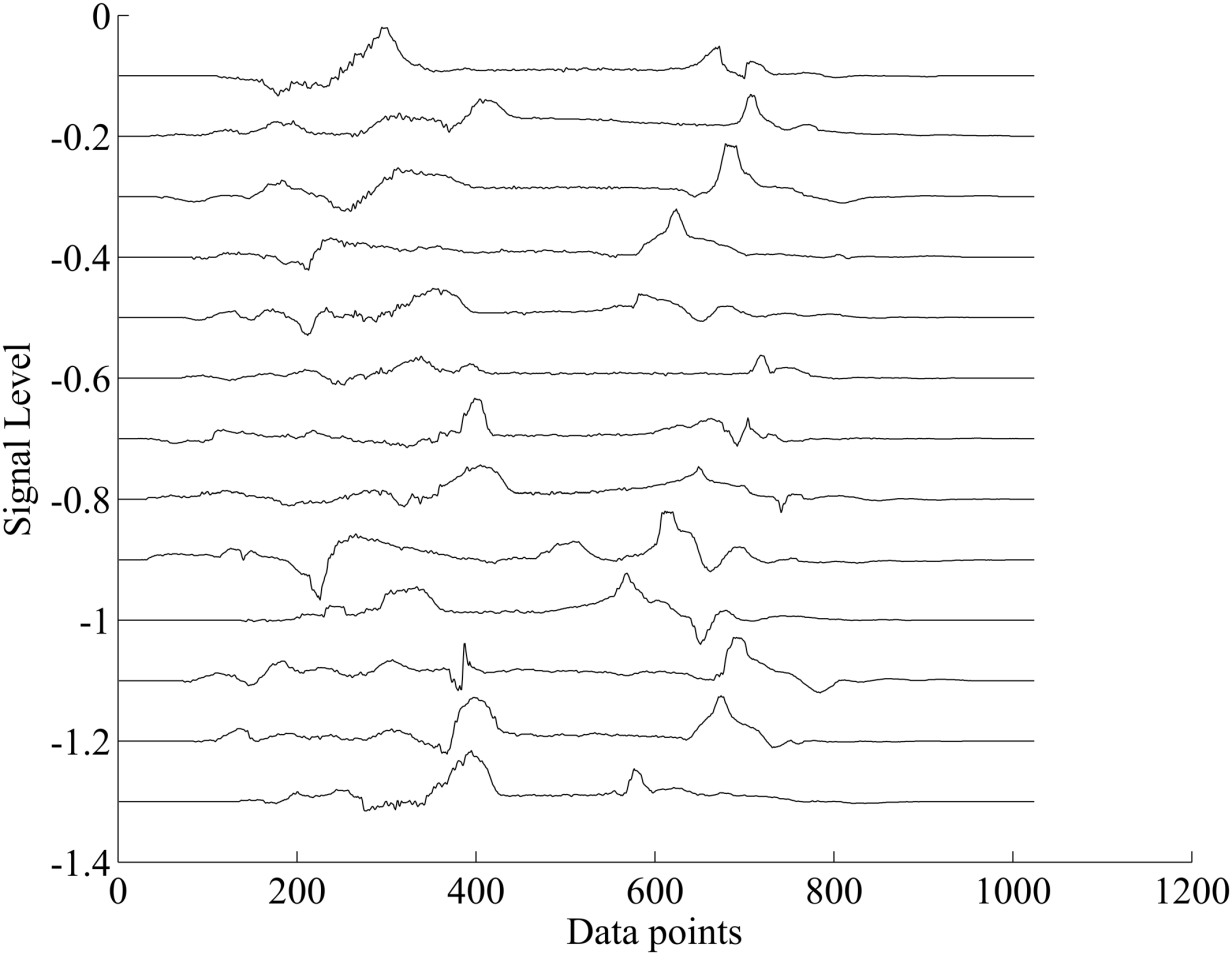
Results of the segmentation for the acceleration waveform of the sand.

**Fig 7:**
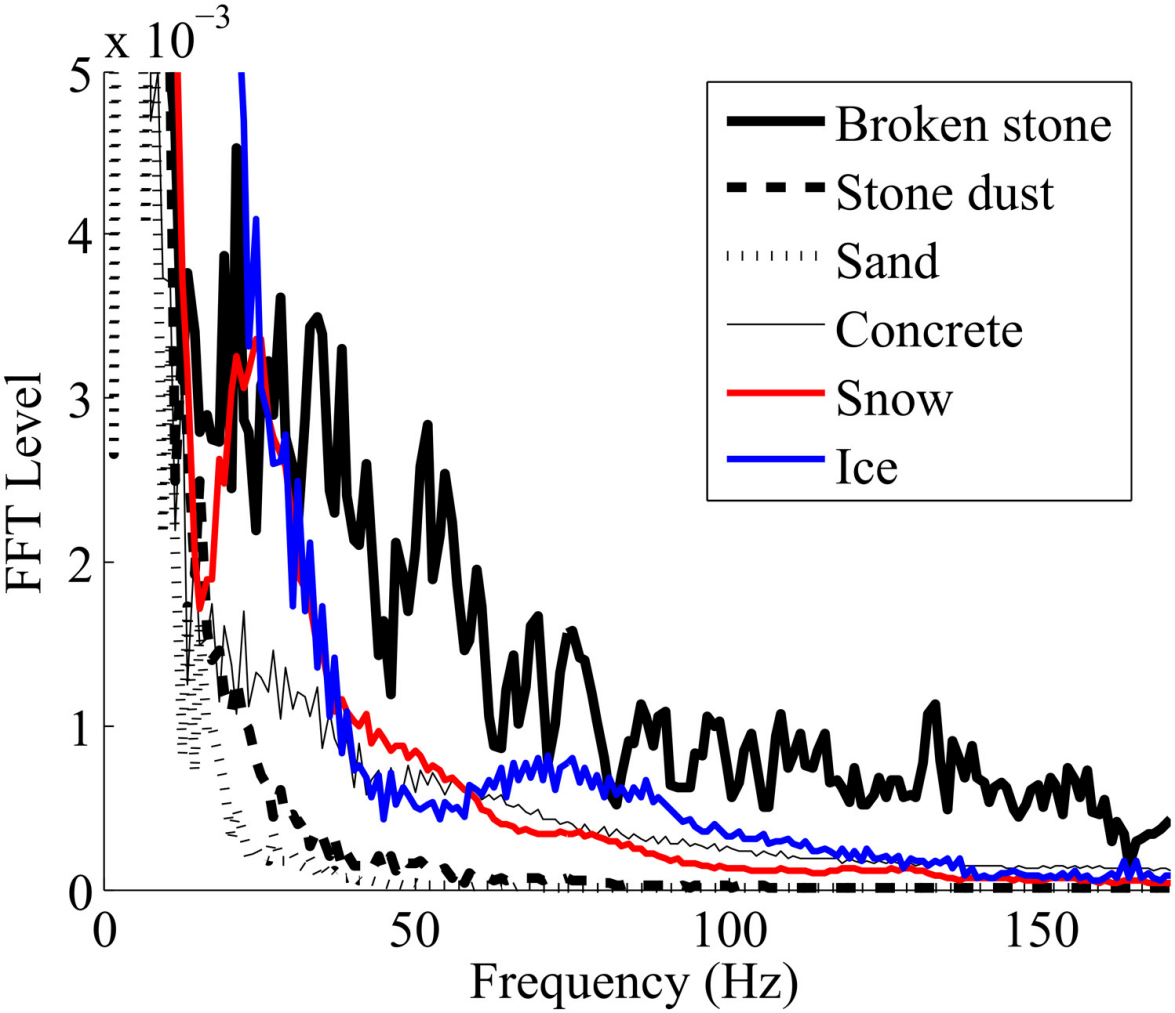
Absolute mean ST-FFT of thirteen acceleration measurements.

1. Acceleration segmentation and preprocessing: Through the previous section, we have noted that waveforms recorded could be associated with physical properties of the soil. To automatically differentiate these waveforms, they are first preprocessed (see Fig 3) throughout a four steps algorithm as shown in Fig 5: identification of the beginning and the end of the heel impact on the soil, zeros padding to obtain **2**^**n**^ data points (for the short-time Fast Fourier Transform or STFT), windowing with a hamming curve and finally filtering with polynomial smoothing filter Savitzky-Golay. The beginning and the end of the heel strike have been found with the FSR force sensor located under the heel. Fig 6 shows the result of the preprocessing for the acceleration segmentation on thirteen steps coming from the interaction with the sand (the offset on each measurement is also applied for Fig 6). For each step, an index is computed for the differentiation of the soil’s physical properties.
2. Differentiation index computation: After the preprocessing, the challenge consists in computing an index that can differentiate the physical properties in real time. For this, we first use Fast Fourier Transform (FFT) to convert each acceleration waveform coming from preprocessing (as shown in Fig 6) from time domain to the frequency domain. Fig 7 gives the mean absolute value of the FFT for one foot contact. Thereafter, we compute the centroid of the spectral response. To avoid computational burden, polynomial center is computed along abscissa and ordinate and then divided by the area of the spectral response. This operation may be labeled spectral centroid and is noted by the coordinate $\hat{x}=\;(S_{x},S_{y})$. The centroid of a set of *n* points masses *m*_*i*_ located at position *x*_*i*_ is computed using:

$$\hat{\text{X}}=\;\frac{{\sum^{\text{​}}}_{\text{i}=1}^{\text{n}}{\text{m}}_{\text{i}}{\text{x}}_{\text{i}}}{{\sum^{\text{​}}}_{\text{i}=1}^{\text{n}}{\text{m}}_{\text{i}}}$$ (1)

$$\hat{\text{X}}=\;\frac{\sum^{\text{​}}{}_{\text{i}=1}^{\text{n}}{\text{x}}_{\text{i}}}{\sum^{\text{​}}{}_{\text{i}=1}^{\text{n}}{\text{m}}_{\text{i}}}\text{with }{\text{m}}_{\text{i}}=\text{m}=1$$ (2)

**Fig 8:**
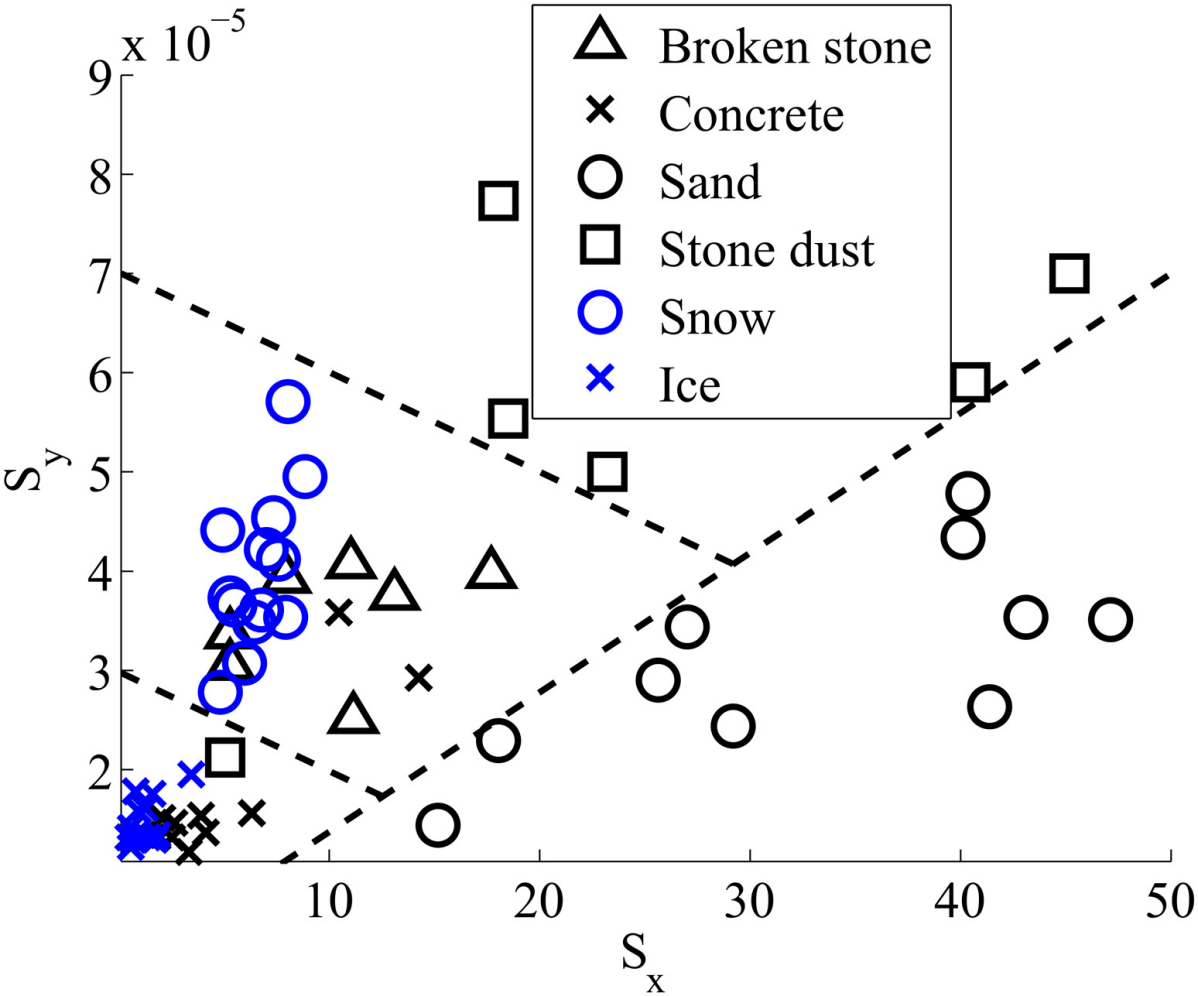
Centroid positions for some signal waveforms frames.

Fig 8 shows the final result of this computation process. Each data point represents one heel strike. It should be noted that a region of exclusion was defined around a mean response to eliminate outliers. The region of exclusion is defined by the boundary of the figure, which corresponds to 50 along the abscissa and 8 x 10^−4^ along the ordinate. As observed in Fig 8, each impact response for a specific soil seems to show a tendency to cluster in a certain region. Using this approach, it has been possible for us to define six clusters to associate each region to a specific soil. Similar analysis using classification techniques was previously done for mobile robotic in [5, 68]. Note that such classification algorithms give a misclassification rate between 1 to 5% in laboratory setup and up to 20% in real conditions.

To improve the detection rate, this paper proposes to use features known in the field of human activity recognition in addition to the previously mentioned FFT centroid approach [69]. These features are, among others, statistical parameters such as mean, standard deviation, variance, and finally the kurtosis function. Computing a level *L* for the soil differentiation is achieved by weighted *W*_*i*_ sum of features *F*_*i*_ as follows:

$$\text{L}=\sum_{\text{i}=0}^{\text{n}}{\text{W}}_{\text{i}}{\text{F}}_{\text{i}}$$ (3)

**Fig 9:**
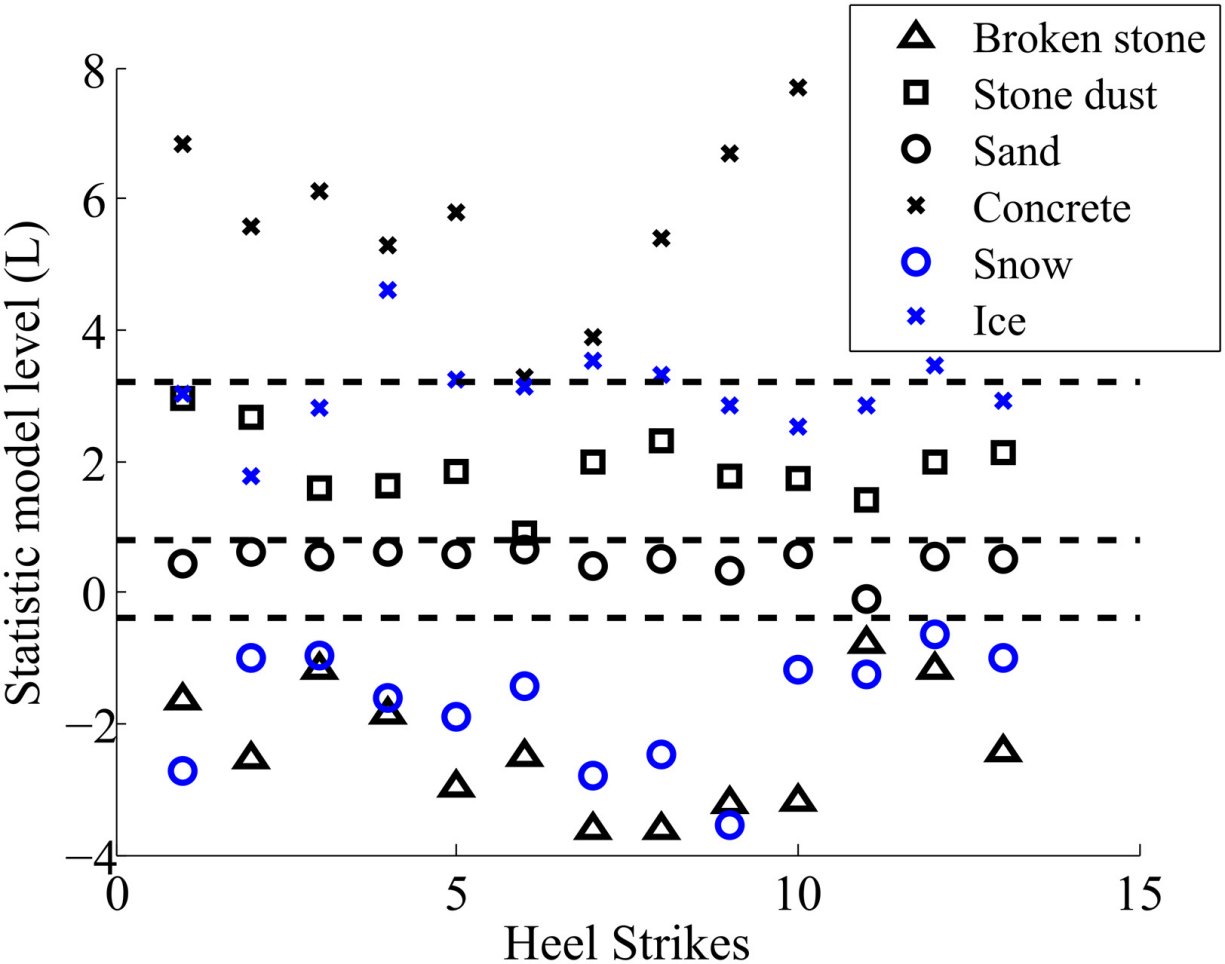
Soil differentiation for each heel strike.

An optimization is performed in order to find the best weight values *W*_*i*_ and the best feature combinations. This optimization is equivalent to what is performed in learning algorithm for artificial intelligence. The optimization tries to increase the distance between each level represented in Fig 9. The threshold for the differentiation is then computed using the distance of the mean value between each given soil.

Six regions were defined from the differentiation method that could correspond to a risk of falling level. We hypothesis that the risk of falling, associated with a type of soil depends on its physical properties. For example, walking on dry concrete correspond to the lowest risk level while walking on soft sand increases the risk to a higher level.

The next section presents the methodology for assessing the risk of falling over different types of soil using a clinical test (the Timed Up and Go test). In this part of the study, the types of soil used are: concrete, parquet, two types of carpet (carpet living room and carpet foam), sand and broken stone. These types of soil are selected because they are frequently encountered in domestic environment.

### Risk of falling evaluation using the enactive insole

All the tests were performed in the Laboratory of Automation and Intelligent 3D Multimodal Interaction (LAIMI) at University of Quebec at Chicoutimi. We present first the population involved in this study. Thereafter, the experimental methodology is detailed.

#### Participants

This study was approved by the local Ethical Committee of University of Quebec at Chicoutimi (certificate number 602.434.01) before the beginning of the measurements. All subjects were first informed about the goal of the study and gave their written consent before participation. Twelve persons with Parkinson’s disease and nine age-matched controls have participated. Persons with PD were recruited from Parkinson Society of Saguenay. Age-matched controls were spouses or kinships of participants with PD. Controls subjects were physically active and without musculoskeletal or neurological disorders. Table 1 presents ages, duration of the Parkinson’s disease, total motor UPDRS, drugs taken and others clinical characteristics. All subjects with PD were scored between 1 to 4 in Hoehn & Yahr scales. PD and controls subjects did not differ by age (*p > 0*.*05*).

**Table 1:** Demographic and clinical characteristics of subjects.

Variables	PD subjects Mean ± SD (Range)	Healthy subjects Mean ± SD (Range)
**No. of subjects**	N = 12	N = 9
**Age (yrs)**	67.7 ± 10.07 (53–77)	66.8 ± 8.0 (57–77)
**Male/Female**	10M /2F	1M / 8F
**Height (cm)**	169.5 ± 21.5	146.6 ± 21.76
**Duration of the disease (yrs)**	10.67 ± 6.05 (1–20)	——
**Hoehn and Yahr scales**	2.5 ± 0.88 (1–4)	——
**Taking of medication**	11/12 (mainly Levodopa)	——
**Total UPDRS score**	43.42 ± 14.9 (16–72)	——
**UPDRS motor score**	20.6 ± 6.5 (9–31)	——
**TUG time in sec (on concrete)**	12.7 ± 1.99 (8–17)	8.9 ± 0.89 (7–10)
**Fear of falling**	33.83 ± 14.75 (16–57)	——
**PDQ-39**	53.58 ± 29.9 (70–116)	——

#### Experimental procedure

**Fig 10:**
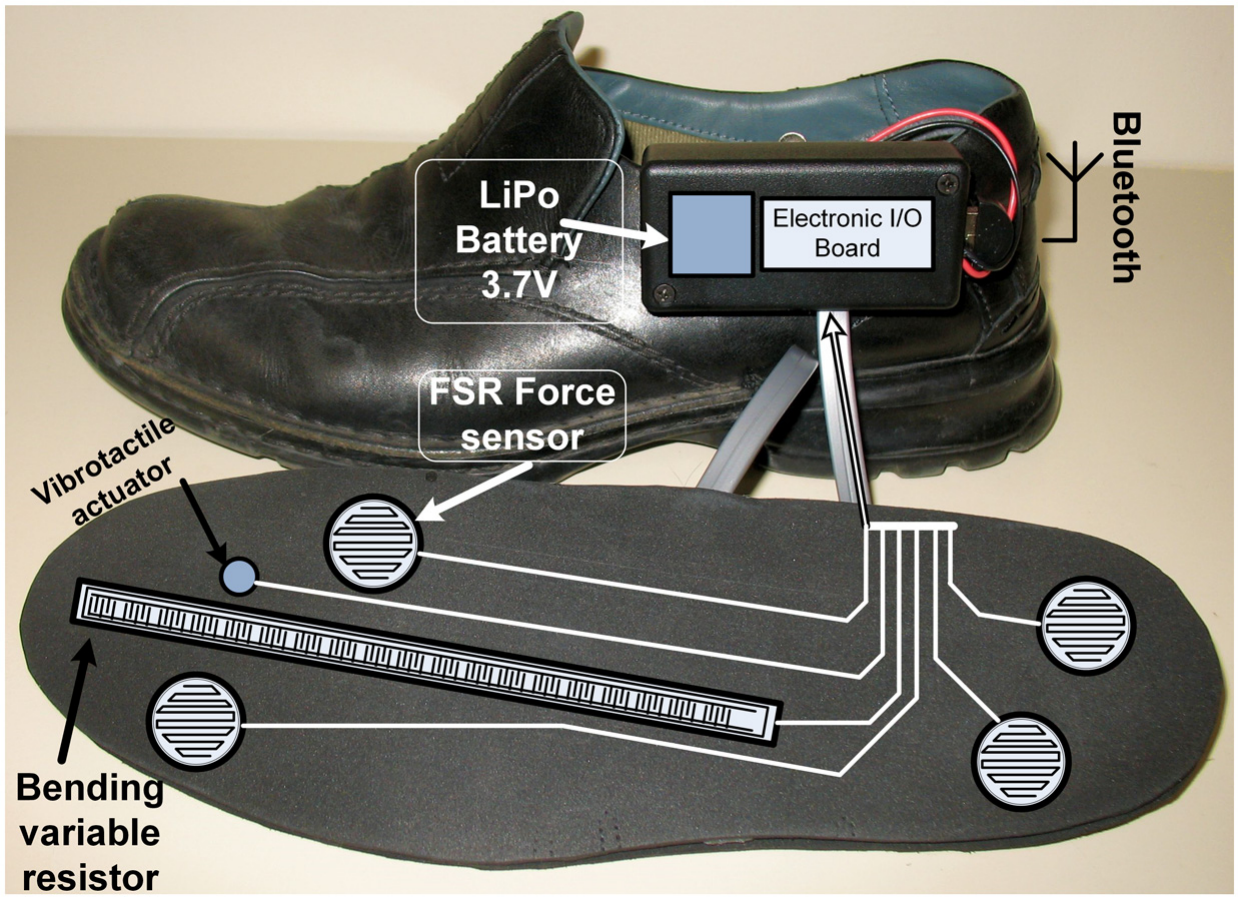
The enactive insole used in the second experiment.

**Fig 11:**
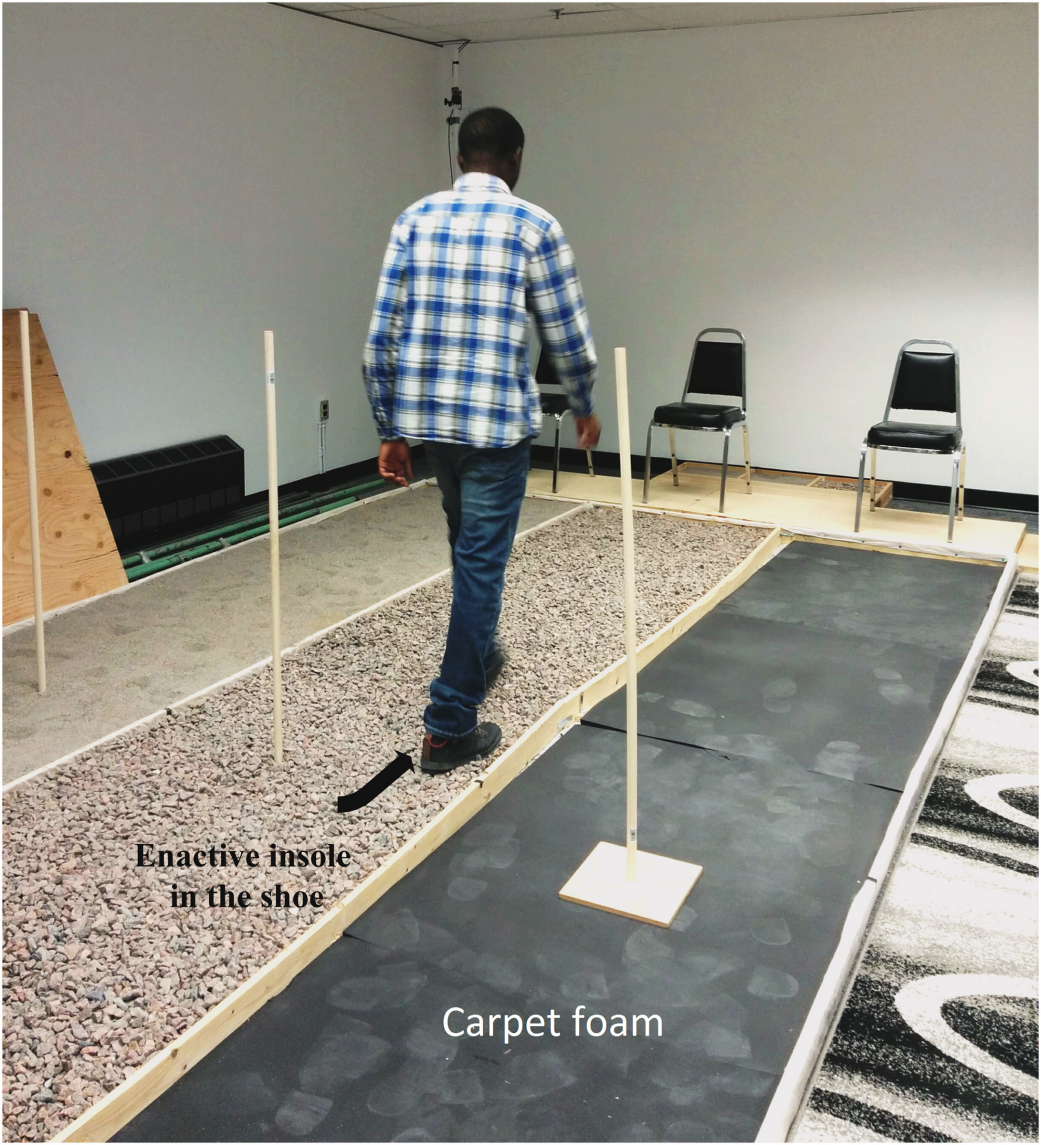
Set up of the experimentation in LAIMI's laboratory.

The procedure was performed as follows:

1. In the first part, the tests with PD subjects were conducted by a physical therapist, (specialized in PD assessment and treatment), one subject at a time, and were constituted of: 1) Unified Parkinson's Disease Rating Scale (UPDRS), 2) Falls Efficacy Scale (FES), an instrument to measure fear of falling, and 3) Parkinson’s Disease Questionnaire (PDQ-39) to assess the quality of life (see the results in Table 1).
2. In the second part, an enactive insole (Fig 10, see also S1 and S2 Figs) was placed in the subject’s shoe. The schematic of this prototype is presented in Fig 2B. The Fig 11 shows the setup of the experimentation. For the subjects with PD, the tests were performed during the peak medication (without presence of freezing of gait). After few familiarization trials, all participants performed the recorded walking trials across three meters. Firstly, the subject was asked to walk along a corridor (Fig 11) by performing the TUG test without cueing. A user-friendly interface was designed on an Android device in order to acquire the cadence of each participant by using the data of FSR and accelerometer sensors. From the two initial and sometimes more baseline walking trials (walking over concrete without cueing), an average value for preferred walking cadence was determined for each individual by the Android application. This was used to calculate the +10% rhythmic vibrotactile cueing frequency. Secondly, participants performed two trials under vibratory stimulation condition at 10% above baseline cadence over each type of soil (concrete, parquet, broken stone, sand, carpet living room and carpet foam) for a total of twenty-four trials for the two conditions. The tactile stimuli were delivered in pulses of fifty (50) ms during the TUG test. Participants were given as much time as they wished to rest between trials, and fatigue did not appear to limit balance and gait control. The presentation order of the type of soil was randomized for each participant. Due to mechanoreceptor dysfunctions, some participants reported being unable to feel the vibrotactile stimulation (one of healthy elerdly and five PD subjects) resulting in the rejection of their results.

#### Computing the risk of falling

Signal processing and the detection of tasks included in TUG test were performed by using an automated algorithm. This section presents first the data acquisition and the TUG signal segmentation. Then the gait parameters computed and the risk of falling assessment are presented.

1. Data acquisition and segmentation of TUG signal: The data of each phase are sent via Bluetooth to the Android application at a rate of 100 Hz in real time. We developed an automated algorithm to segment the phases of the TUG test: 1) the beginning of the test which is coincident with the beginning of the sit-to-stand phase; 2) the beginning of the walking phase, which is coincident with the end of the sit-to-stand phase; 3) the turning phase; and the end of the walking back phase, which is coincident with the beginning of the stand-to-sit phase; 4) the end of the test, which is coincident with the end of the stand-to-sit phase.
2. Gait parameters: After the segmentation, the sensor waveforms allow the detection of the number of steps performed by each participant. The number of steps performed was determined by taking the walking portion of the TUG test. The walking portion is from the end of the sit-to-stand task until the start of the stand-to-sit task. It is known that during walking, a specific pattern of acceleration signals is repeated in each step. The number of steps was taken as the number of peaks in the acceleration signal [70]. Steps counted in acceleration signals were validated against the steps counted from the FSR sensors signals. After the step detection, the algorithm calculates all gait parameters needed in the risk of falling computation. The gait parameters computed in this study have been chosen according to the most used in the literature [71–74] which are: walking speed, instantaneous cadence and stride length. The stride length (*SL*) was determined using the relation suggested in [75, 76]:
  $$\text{SL}=0.98*\sqrt[{3}]{\sum_{\text{i}=1}^{\text{N}}{\text{a}}_{\text{i}}/\text{N}}$$ (4)
  Where *N* is the number of samples, *a*_*i*_ is the mean of acceleration during the time of the stride. The stride length is calculated using the filtered signal and can be estimated as a distance travelled during the test.
3. Risk of falling computation: In order to estimate a risk of falling in uncued or vibrotactile condition, we used the Coefficient of Variation (CV) of each instantaneous gait parameter as suggested in [77]. This coefficient described by Gabell and Nayak [78] is expressed by:
  $${\text{CV}}_{\text{j}}=100*\frac{{\text{σ}}_{\text{j}}}{{\text{M}}_{\text{j}}}$$ (5)
  where *σ*_*j*_ and *M*_*j*_ are respectively the standard deviation and the mean of the gait parameter *j*.

It is known that a faller exhibits a greater Coefficient of Variation (CV) than a non-faller or compared with a young adult [71, 79]. Indeed, musculoskeletal deficits or Parkinson’s disease causing irregular gait may explain the greater coefficient of variation observed among fallers. Also, we note that a greater dispersion in the gait parameters corresponds to a greater coefficient of variation. Finally, we have computed a risk of falling based on the results suggested by Noshadi et al. [80] for instability assessment. For better clinical assessment tests, the goal was to combine the most significant gait parameters into a single score. The proposed risk of falling is expressed in Eq (6).

$$\text{Risk}=\text{α}*\left({\text{CV}}_{\text{cad}}+{\text{CV}}_{\text{SL}}\right)$$ (6)

Where *α* represents the coefficient attributed to the variability of the gait feature (cadence and the stride length). This coefficient can be set by physicians, clinicians and domain export to tailor the instability assessment to best fit the individual patient [11]. In our study, this coefficient is inversely proportional to the walking speed value (the value without unit). CV_cad_ and CV_SL_ are respectively the Coefficients of Variation of cadence and the stride length.

#### Human perception of the risk of falling

After the TUG test, each participant was questioned about the type of soil that induced a perception of risk of falling while walking in the uncued situation.

#### Statistical methods

Data analysis was performed using the software PRIMS-5 by Graph Pad Co San Diego USA including descriptive statistics. The risk of falling and TUG time (dependent variables) in uncued and rhythmic vibrotactile conditions were analyzed using a one-way analysis of variance (ANOVA 1) for each group (PD subjects, control) across the types of soil (the independent variable). A student “t” test for independent samples was used to compare the two groups. The risk of falling was also compared between groups and across conditions (with and without cueing) using two-way analysis of variance. Pairwise comparisons identified significant differences between conditions, and Bonferroni corrections were used during all analyses. Statistical significant was set at the 95% confidence level (*p* < 0.05).

## Results and Discussion

**Fig 12:**
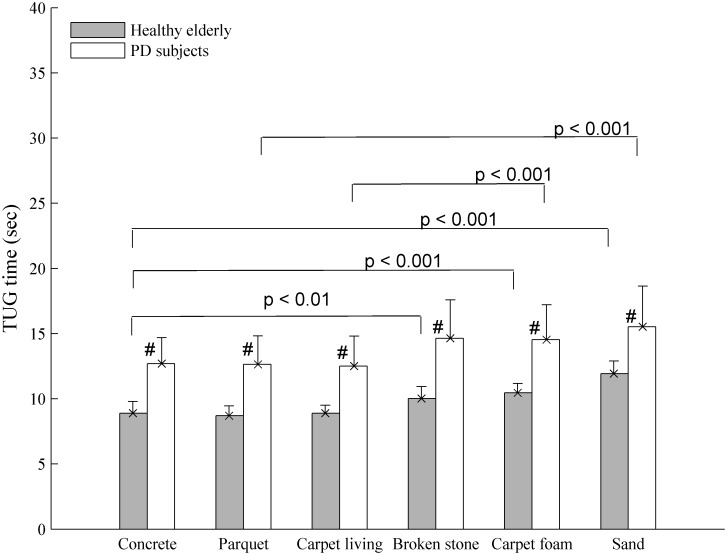
TUG time (mean ± SD) among PD and healthy subjects over different types of soil in uncued condition. Note: # denotes a significant difference between the PD and control groups. The p-values (p < 0.01) indicate a significant difference between the types of soil for the PD participants or controls subjects.

**Fig 13:**
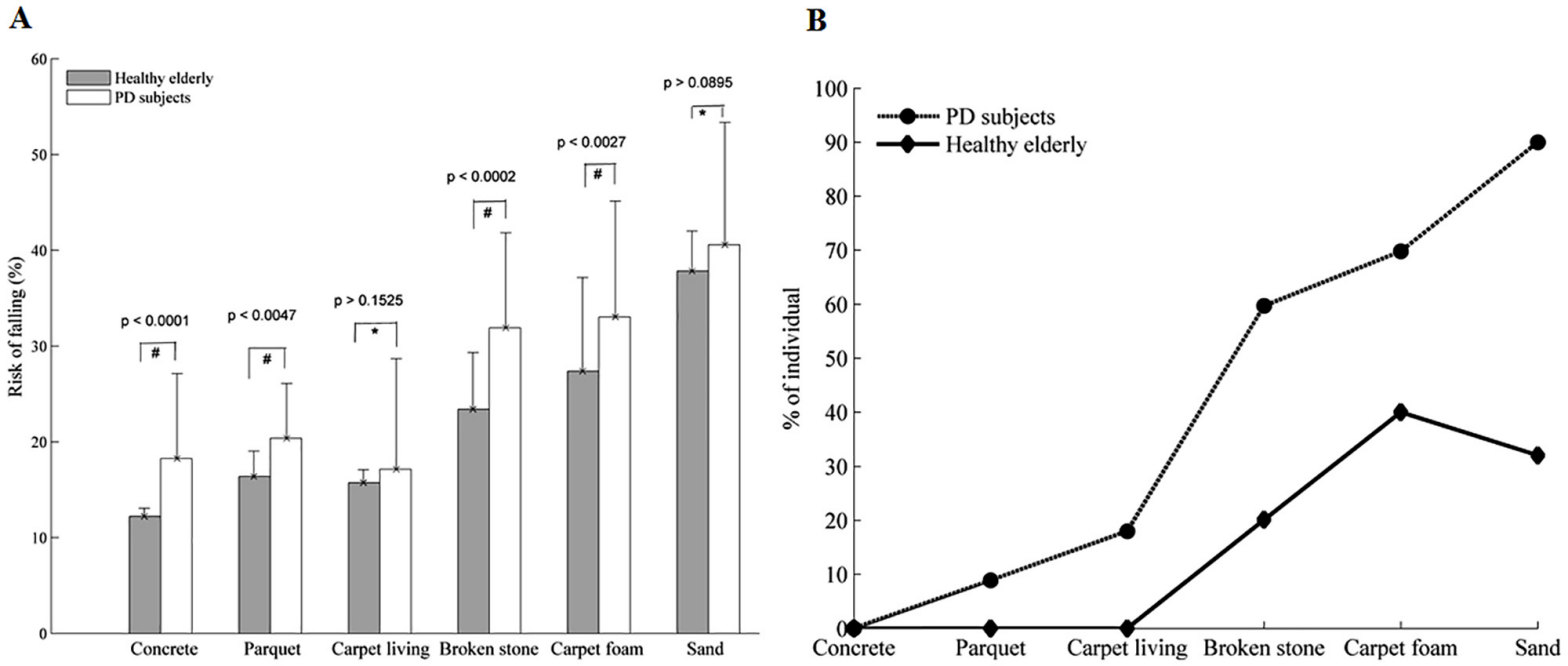
Risk of falling (mean ± SD) over each type of soil in uncued condition. (A) from gait parameters. (B) from questionnaire: percentage of participants who perceived a risk of falling. Note: # denotes a significant difference between the PD and control subjects and, * denotes a non-significant difference between the two groups.

**Fig 14:**
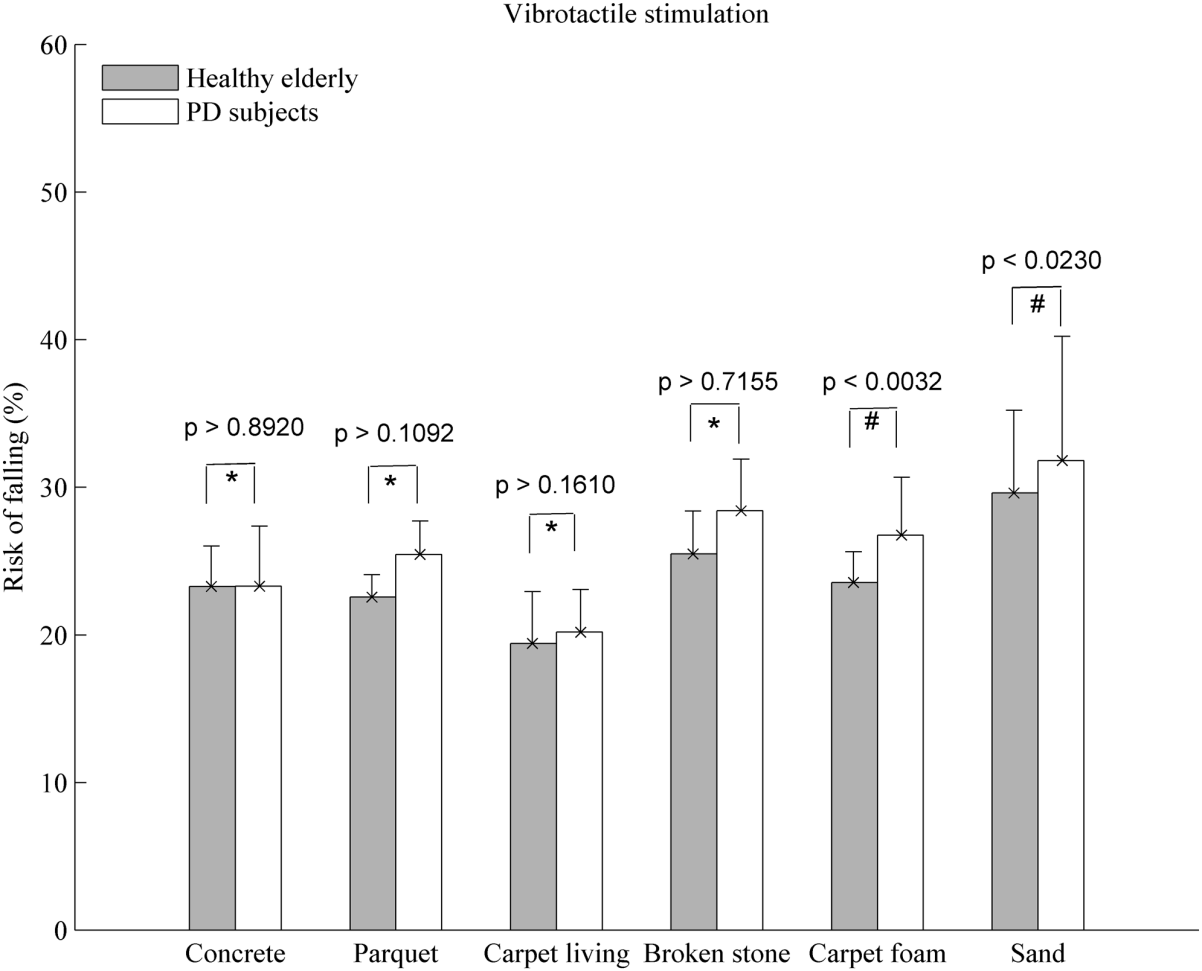
Risk of falling (mean ± SD) from gait parameters over each type of soil in the vibrotactile condition (seven PD subjects and eight healthy elderly). Note: # denotes a significant difference between the PD and control subjects and, * denotes a non-significant difference between the two groups.

In all figures below, the mean values are reported. The errorbars indicate the standard deviation (SD). As shown in Figs 12 and 13A, increased TUG time and risk of falling were observed among the PD subjects compared to controls subjects in the uncued situation. For the TUG times, the t-test showed a significant difference between these two groups (p < 0.05). In the most types of soil, a significant difference was found as far as the risk of falling is concerned (Fig 13A, p < 0.05) except over the sand and carpet living room where p > 0.05. In rhythmic vibrotactile condition (Fig 14), by using the t-test between the two groups, no significant difference was found in the most types of soil for the risk of falling (p > 0.05) except over the sand and carpet foam. However, a decrease in the risk of falling level has been observed over these two types of soil (as shown in Table 2), meaning that our suggested system could help reducing the risk of falling compared to the uncued situation in Fig 13A. Moreover, the two-way analysis of variance is performed in order to detect any interaction effect between groups and conditions (with and without cueing). It shows that the difference in means of the two conditions was significant at 0.05.

**Table 2:** Mean ± SD of risk of falling over different walking environments.

Conditions	without cueing (%)		with cueing (%)	
	Healthy elderly	PD subjects	Healthy elderly	PD subjects
Concrete	12.21 ± 0.85	18.25 ± 8.85	23.29 ± 2.72	23.30 ± 4.08
Parquet	16.39 ± 2.63	20.37 ± 5.72	22.56 ± 1.51	25.45 ± 2.27
Carpet living room	15.74 ± 1.35	17.14 ± 11.53	19.42 ± 3.51	20.18 ± 2.90
Broken stone	23.41 ± 5.9	31.89 ± 9.93	25.49 ± 2.89	28.41 ± 3.48
Carpet foam	27.37 ± 9.78	33.04 ± 12.09	23.55 ± 2.08	26.76 ± 3.89
Sand	37.83 ± 4.17	40.57 ± 12.78	29.59 ± 5.60	31.79 ± 8.41

This study demonstrates the possibility of using vibrotactile cueing to reduce the risk of falling in elderly while walking in unknown environment. But first, we have differentiated using an algorithm the types of soil in the walker's environment.

### Discussion on the type of soil differentiation

Looking at Fig 8, we observe that some of the stone dust spectral centroids are situated outside of their associated cluster. One may note that this soil, is the most deformable among the six types experimented. Considering that the response of soil vibrations depends on the force applied by the foot, we suppose that the soil deformation changes the foot orientation and thus changes the force distribution under the foot. Similar observations were found in [15]. This variation in the force distribution generates an unpredictable response of the soil. It is therefore possible that several spectral centroids are found outside their respective cluster. In order to improve these first results, some features computations are incorporated to the algorithm. The results shown in Fig 9 demonstrate an accurate differentiation of the soil physical properties. Our results show, with ST-FFT centroid alone, a detection rate of 77% and are improved to 99% when adding level *L* coming from the weighted sum of the features.

### Discussion on the computed risk of falling

PD subjects have a greater TUG time and risk of falling than the controls subjects in the uncued condition (Figs 12 and 13A). This result is consistent with our expectations. This is explained by the quality of afferents of sensorimotor stimulation or the Parkinson’s disease process causing irregular gait and a greater coefficient of variation. Also, the soil physical properties increase the dispersion in the coefficient of variation of gait parameters and then increase the risk of falling. Thereby, the effect of rhythmic vibrotactile cueing may be beneficial as suggested by Galica et al. [44]. Looking at Fig 14, we can show that rhythmic vibrotactile applied to the soles of the feet during the gait cycle in the TUG test can reduce and regulate the gait variability and then the risk of falling in unknown environment. Similar observations were found in [44, 71, 81] but with one type of soil or by using another cueing such as auditory and/or visual. The increase risk of falling observed over the rigid surfaces (Table 2, Fig 14), compared to uncued condition (Fig 13) can be explained by the 10% above the cadence used. This may be increased the risk of falling in these surfaces. However, it has been demonstrated in literature that an appropriate frequency of stimulation can improve gait parameters in PD subjects [44, 73, 82] and then can be useful in perturbed environment as shown in this study (Figs 13A and 14).

The risk of falling obtained from PD and controls subjects has been divided into six groups corresponding to the six types of soil. One-way Analysis of Variance (ANOVA) was performed in order to compare level of stability. The ANOVA results are reported as an F-statistic with its associated degrees of freedom and p-values. The null hypothesis *H*_*0*_ is that all the means of risk of falling from the six different types of soil are equals. This analysis of variance leads to the conclusion that there is a significant effect related to the type of soil on the risk of falling in uncued condition (*F (5*, *48)* = 31.11, *p* = 5.68 x 10^−14^ < 0.05, for controls subjects) and (*F* (5, 66) = 40.25 *p* < 0.05, for PD subjects) contrary to the cueing condition. Pairwise comparisons using Tukey’s HSD post-hoc tests showed no significant effect between the three rigid surfaces (Concrete, Carpet living room, and Parquet). However, a significant effect of type of soil was found by comparing concrete and broken stone (*p* < 0.01); concrete and carpet foam; concrete and sand; parquet and sand; carpet living room and carpet foam with *p* < 0.001 in each comparison.

No significant difference was found over the sand and carpet living with the t-test performed between the two groups (Fig 13A). This can be explained by the fact that the sand affects both healthy and PD subjects. Also, these two groups (healthy and PD subjects) pay more attention when walking over the sand. Thus, the walking test on deformable soils, probably on sand, can represent an excellent model to detect the risk of falling in healthy elderly (HE) and PD’s subjects. As for the carpet living room, even if we do not have a significant difference between the two groups, the standard deviation is different. This shows an effect of this type of soil on the walking of PD subjects.

Fig 13B shows the percentage of participants who perceived a risk of falling over each type of soil in the uncued condition. The sand was the surface on which PD and control subjects had the most difficulties walking on. A difference more than 40% was found among PD subjects as shown in Fig 13B. This can be explained by the lack of proprioception among the PD subjects and by the fact that the sand was the soil that produced the most fear of falling in the two participants groups. However, it was the carpet foam that threatened the most balance during walking (Fig 13B). This could be explained why a non-significant difference was found between PD and controls subjects in the vibrotactile condition on these two types of soil (Fig 14). Indeed, the difficulties to maintain balance over these types of soil would be affected the rhythmic walking of certain participants. However, the effort to regulate the participant’s walking pace using vibrotactile stimulation has decreased the risks of falling over these soils (Table 2). The two-analysis of variance indicated that there was also an interaction effect of group and condition (with and without cueing) for the risk of falling over one of the rigid surface: concrete (F = 7.155, p = 0.012) and one of the deformable soils: gravel (F = 9.414, p = 0.004). These results indicated that the groups used the conditions differently. However, it was not the case of the other types of soil indicating no interaction between the groups and condition. The risk of falling computed using the TUG test over different types of soil is used to adjust the risk level for each cluster presented in Fig 9.

### Limitations of this study

The limitation of this study is the generalization of finding to a wider population due to small sample size used. In other words, the limitation of this work is the fact that some participants did not feel the vibration signals. This is probably due to aging mechanoreceptors or the duration of the Parkinson’s disease. Therefore, the vibrotactile cueing must be improved by increasing amplitude of vibrations for a better feeling and reducing perceptual conflict with the soil vibration at each heel strike. Thus, our next generation of enactive shoe will employ a new generation of flat voice-coil actuators, able to significantly increase the produced vibration amplitude and frequency waveforms (stimulation of different mechanoreceptors). Given that the fall is a multi-factorial phenomenon, the other limitations of this study are the combination and the generalization to all gait measures in a single index. Our next paper which includes several gait measures using an artificial neural network (ANN) will take this into account. The design of such an algorithm for more gait features and for all activities is still an undergoing issue. However, our first evaluation shows encouraging results.

## Conclusion and Future Work

This paper has addressed not only the automatic discrimination and differentiation of soils by their acceleration response (vibration) to a heel strike but also the risk of falling over several types of soil. Through an analysis of the frequency domain and the computation of a spectral centroid, we determined an index that helps at differentiating selected types of soil, which is one of the major factors involved in the risk of falling. This approach has been adopted since it can be easily implemented on a microcontroller. We found that our suggested enactive insole could help to reduce the risk of falling using rhythmic vibrotactile cueing while walking over different types of soil. Finally, the TUG experiment shows that we can successfully associate a risk level for each type of soil that was tested. Balance control is not only improved by rhythmic stimulation but also is moderate over the types of soil. This work should be included in a more complex strategy for avoiding fall. Therefore, other algorithms such as the k-Nearest Neighbor (kNN), support vector machine (SVM), principal component analysis and an artificial neural network are currently under investigation since we are interested in the development of better on-site assistance for gait disorder analysis and for long term monitoring of persons in loss of mobility. For example, the enactive insole will need a complementary user-friendly serious game in order to train balance each day. Moreover, since rhythmic vibrotactile cueing shows a reduction in the risk of falling, in a near future, further experimentations will be conducted using other stimulations.

## Supporting Information

S1 Fig The enactive insole used for the men. (TIF)

S2 Fig The enactive insole used for the women. (TIF)
