# Supplementary figures and images for: Use of an Enactive Insole for Reducing the Risk of Falling on Different Types of Soil Using Vibrotactile Cueing for the Elderly
Source: PLoS One. 2016 Sep 7;11(9):e0162107. doi: 10.1371/journal.pone.0162107 (PMC5014342; doi:10.1371/journal.pone.0162107)

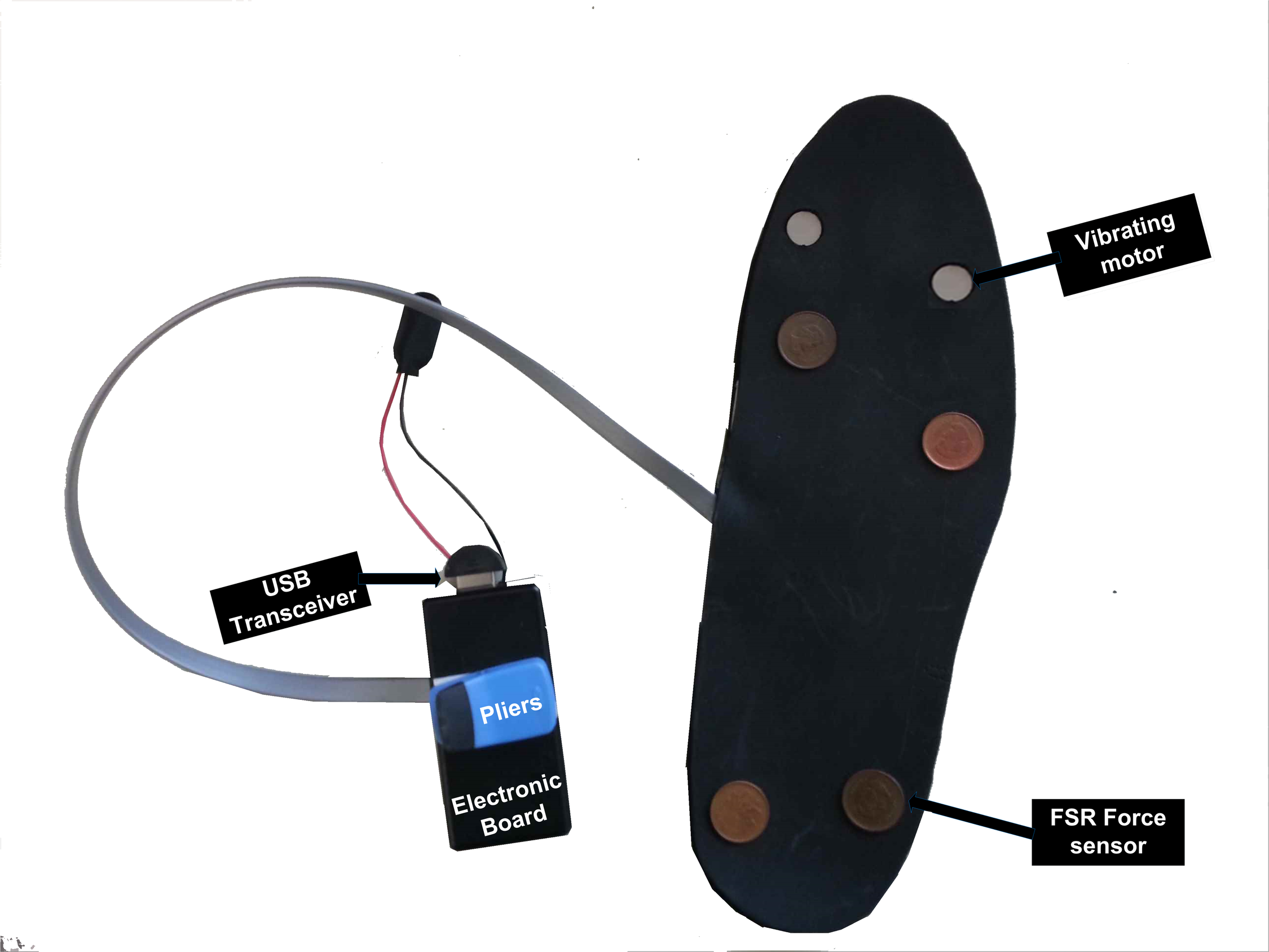

Supplement: S1 Fig — (TIF) [file pone.0162107.s001.tif]

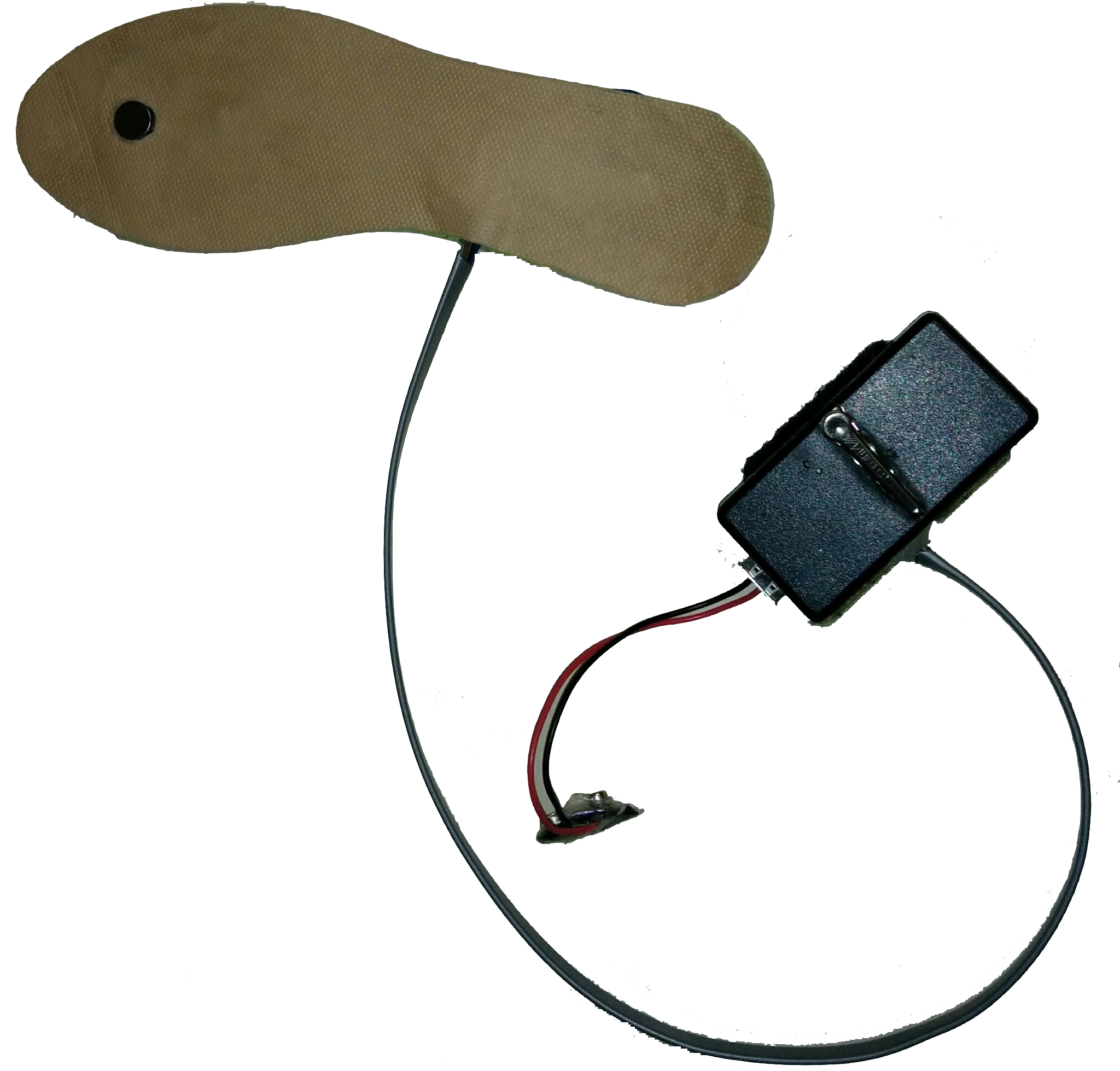

Supplement: S2 Fig — (TIF) [file pone.0162107.s002.tif]
